# Digital twins for managing bridge climate change adaptation

**DOI:** 10.12688/openreseurope.17809.1

**Published:** 2024-08-08

**Authors:** Sakdirat Kaewunruen, Hao Fu, Adefolarin Adebiyi, Pasakorn Sengsri

**Affiliations:** 1 University of Birmingham, Birmingham, England, UK; 2 Department of Highway, Ministry of Transportation, Department of Highway, Bangkok, 10400, Thailand

**Keywords:** Building Information Modeling, Digital Twins, Bridge, Climate Change, Adaptation Measures

## Abstract

**Background:**

Bridges are vital construction infrastructures that almost every nation needs in order to function. Climate change is a significant issue, that especially affects the construction industry. It is very important that bridges are able to withstand the impacts of climate change and adaptation measures will be required to achieve this.

**Methods:**

The digital twin will be developed using BIM to manage the climate change adaptation measures for the bridges. A 6D BIM model will be created that includes the 3D Revit model of a bridge featuring climate change measures, the climate change adaptation measures timeline schedule, climate change adaptation cost estimation, and carbon emission estimation, which will be produced using Revit software, Navisworks, and Granta EduPack. The results will show how 6D BIM can be used to support the adaptation of bridges to the effects of climate change.

**Results:**

The findings underscore the efficacy of 6D BIM in enhancing bridge resilience against climate change impacts. The 3D model demonstrates integration of adaptation measures without compromising bridge functionality. Moreover, the 4D model's timeline scheduling facilitates hazard anticipation, project planning, communication enhancement, collaborative efforts, and project visualization. Cost estimations from the 5D model reveal varying costs among adaptation measures, while the 6D model highlights differences in carbon footprints. These BIM dimensions enable stakeholders to analyze effects on project costs and energy consumption, aiding sustainability and cost-efficiency considerations.

**Conclusions:**

The study exhibits the literature review analysis, the risk assessment, research on climate change adaptation strategies, and implementation using Revit 2022, Navisworks 2022, and Granta EduPack software. By contributing to the adaptation of bridges to climate change effects, the research has provided valuable insights and practical implications for enhancing bridge resilience globally.

## Plain language summary

Bridges are vital construction infrastructures that almost every nation needs in order to function. Climate change is a significant issue, that especially affects the construction industry. It is very important that bridges are able to withstand the impacts of climate change and adaptation measures will be required to achieve this. The digital twin or virtual replica of the bridge will be developed to manage the climate change adaptation measures for the bridges. A 6D BIM model will be created that includes the 3D Revit model of a bridge featuring climate change measures, the climate change adaptation measures timeline schedule, climate change adaptation cost estimation, and carbon emission estimation, which will be produced using Revit software, Navisworks, and Granta EduPack. The results will show how an interactive 6D BIM (transforming into a digital twin) can be used to support the adaptation of bridges to the effects of climate change. Our study contributes to the adaptation of bridges to climate change effects, the research has provided valuable insights and practical implications for enhancing bridge resilience globally.

## Introduction

A bridge is a structure with the purpose of carrying vertical loads, that are usually in motion, while spanning between supports horizontally. It is vital that both the supports and span are strong enough to carry out their roles ^1^. Bridges are a very important part of a nation’s infrastructure as they allow raw materials and finished goods to be shipped to factories, warehouses, suppliers, distributors, shops, and customers. When a bridge is no longer in use, economic activity reduces or could come to a complete stop ^2^. Bridges can support integrated transport networks in becoming more sustainable while also improving the health, physical condition, and wellbeing of the public ^3^.

**Figure 1.**
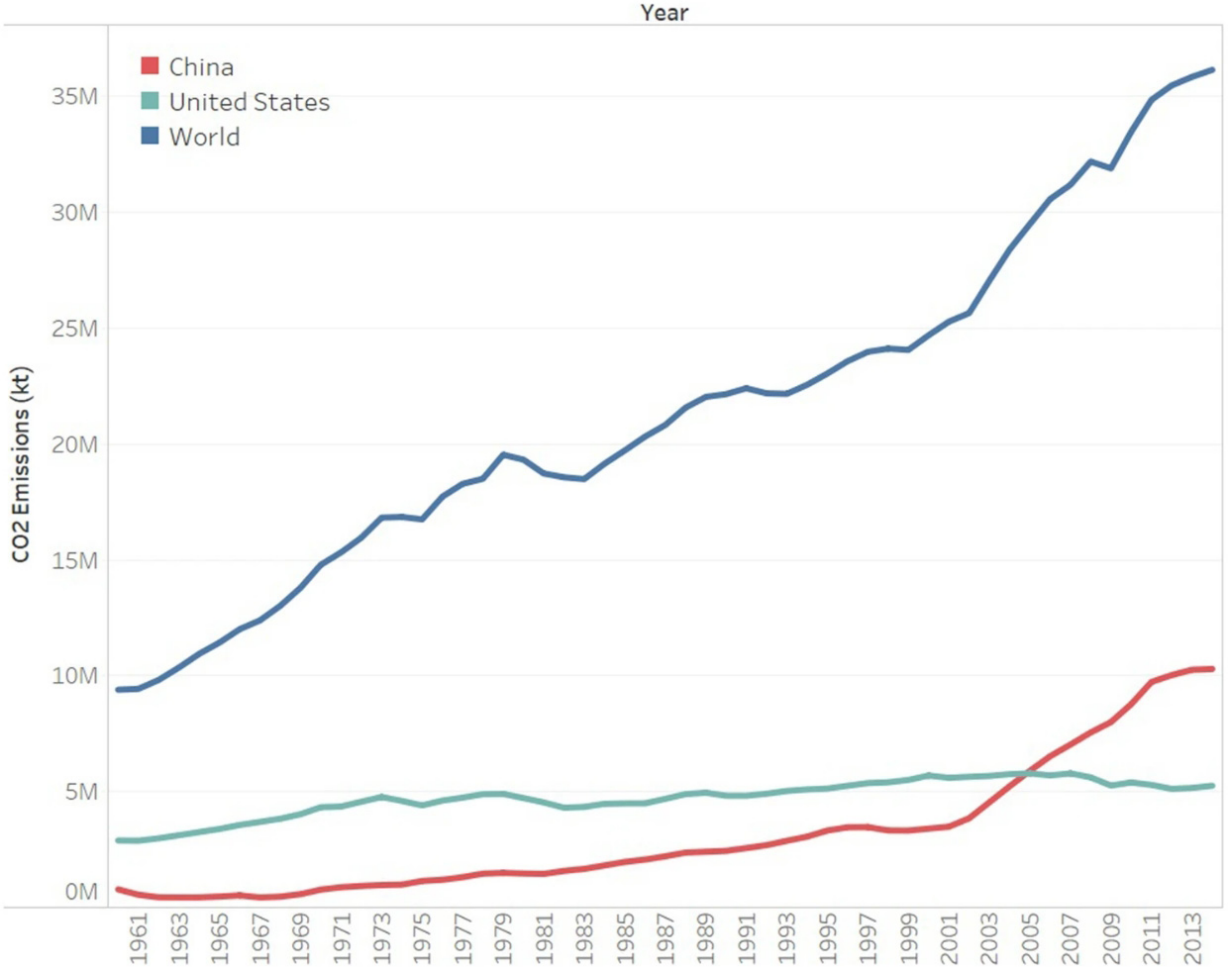
CO2 emissions across the globe and, in China, and the United States. Reused with permission from 5.

Over the past 50 years, global warming observed has occurred mainly due to human generated emissions of gases that trap heat. These emissions are mostly produced from burning fossil fuels, while the clearing of forests and agricultural practices also largely contribute to global warming ^4^, as the historical carbon emission shown in Figure 1 by 5. Climate change is causing an increase in the pollution of water and air, greenhouse gas emissions, eutrophication, massive land conversion, that will all have devastating and amassing effects ^6^.

Due to their significantly long service life, it is extremely important to ensure that bridges are reliable against the impacts of climate change. Climate change is expected to cause the frequency and intensity of certain extreme events (such as floods) to grow, therefore bridges must still be able to function during these events and rapidly recover afterwards, meaning they are very relevant to the impacts of climate change ^7, 8^.

The bridge that will be used for this research is a 3D model of a bridge previously created by 9 on Revit 2022 Software that is a standard 9m roadway width, slab bridge. It is assumed that the bridge is located in the UK, therefore its weather conditions will be considered. It is assumed that the bridge has not yet been constructed, so adaption measures can be implemented during the process of the construction of the bridge. It is assumed that the bridge is located by the sea which will be considered when researching the adaption measures.

The primary goal of this study is to develop a comprehensive 6D Building Information Modelling (BIM) framework that includes a 3D Revit model of a bridge with climate change mitigation measures, as well as a detailed timeline schedule for implementing adaptation strategies, cost estimates for climate change adaptation measures, and carbon emissions assessments. The BIM framework will be built with powerful software tools including Revit, Navisworks, and Granta EduPack.

The paper aims to investigate the following aspects: firstly, to ascertain the vulnerabilities posed to bridges by climate change; secondly, to explore and evaluate various climate change adaptation strategies conducive to mitigating the adverse impacts on bridges; thirdly, to devise a BIM system capable of facilitating the assessment of costs, carbon emissions, and temporal resources associated with implementing climate change adaptation measures for bridges; and finally, to analyse and deliberate upon the outcomes derived from the deployment of the 6D BIM model, thereby elucidating the prospective efficacy of climate change adaptation measures in the context of future developments.

## Literature review

### BIM in the construction industry

BIM technology is considered to be one of the greatest tools for the improvement of management systems in Architecture, Engineering, Construction, Owner, and Operator (AECOO) industries ^10^. For the advantages of BIM, there are mixed opinions and perspectives, which leads to a widespread misunderstanding of the outcomes that are expected from BIM. The collaborate process that happens between stakeholders in a project is prevented due to this issue. This means that, when BIM is being discussed, it is essential to be precise, which will lead to misunderstandings being avoided and making sure that stakeholders are able to deliver the work that has been demanded concerning BIM ^11^. The most common benefit of BIM that is reported relates to the control that is had throughout the life cycle of the project, reduction of cost, and the substantial amount of time being saved. For the negative aspects of BIM that are reported, the main issue was the use of BIM software. Raising awareness, training and education are vital ways of dealing with the problems of using BIM ^12^.

Research for establishing BIM into the infrastructure industry, such as the building industry, has recently been undertaken. One of BIM’s many advantages is the lifecycle management of infrastructure, starting from planning and design to construction and maintenance. Introducing the BIM process at the planning stage will thoroughly improve project management and collaboration between the stakeholders of the project ^10^. Furthermore, BIM does not just support collaboration in the design stage, but also helps improve the quality of the design and reduces errors. The BIM process can help increase productivity and workflow during the construction stage by decreasing the wastage resources and time. BIM data on infrastructure that has been stored during the planning and design stage of the project can also be used later for maintenance. Additionally, maintaining the infrastructure in an effective way can be achieved through the integration of BIM with the latest modern technologies, for example, unmanned robots and Unmanned Aerial Vehicle (UAV) systems that have digital cameras or 3D Laser Scanning and Light Detection and Ranging (LiDAR), artificial intelligence algorithms, and 3D model construction algorithms.

**Figure 2.**
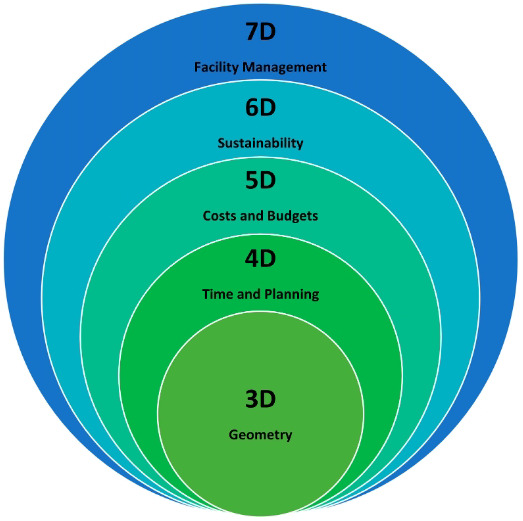
Diagram showing the BIM levels from 3D - 7D. Reused with permission from 13.

Figure 2 shows different BIM levels from 3D to 7D ^13^. The 6D BIM dimension is used to analyse the energy consumption of a building and produce energy estimates. Therefore, sustainability performance information is given during the design’s initial stages ^14^. Furthermore, 6D BIM is also called integrated BIM because of the detailed information that helps operations and facility management. It includes information such as the component’s manufacturer and installation date, to ensure better performance. Therefore, the energy requirement and decommissioning information of the building is used to align the performance of the building ^15^.

### Types of bridges

For a more modern type of construction, It was suggested by 16 that any urban or rural area can be decorated by a suspension bridge that has overhead cables that supports its roadway. They are aesthetically pleasing, light in weight and are capable of spanning longer distances than any other type of bridge. A composite truss bridge with hollow structural section members is considered to be the structure that is suitable for large-span bridges and bridges with heavy load ^17^. Concrete-filled steel tub arch bridges are an exceptional type of steel-reinforced concrete composite bridge, where the steel tube has its local stability enhanced when it is filled with concrete, while the strength and toughness of the concrete are enhanced because it is confined by the steel tube ^18^. Cable-stayed bridge are built with three main structural elements, which are towers, cable-stays, and the deck. Several inclined cable stays are used to support the deck along the length of the bridge. Therefore, large-span bridges can be built with shallow decks ^19^. Cantilever bridges are built through a method of progressive construction of a cantilever that is done in segments, then they are stitched to segments that were casted by prestressing beforehand ^20^. A steel–concrete hybrid beam bridge is a bridge in which steel beams replace the mid-span concrete beams. This will lower the weight of the bridge. This type of bridge has many advantages, making it more popular in practical engineering ^21^. It is beneficial to consider the different types of bridges that are being constructed to help develop the potential climate change adaptation measures.

### BIM application for bridges

In the near future, BIM will be available for recently built bridges and existing bridges. BIM models are able to and will be incorporated into Bridge Management Systems and will therefore considerably improve the quantity of information that is valuable in future Bride Management Systems. Other than accurate spatial and semantic specification, BIM is also capable of embedding realistic structural system of a bridge along with the applicable load situations ^22^. For years, the building industry has gained a lot from the advantages of BIM including information modelling and digital data exchanges that occur between various parties that participate in the planning, design, fabrication, and construction of the project. In order to also gain the proven advantages, the bridge industry have recently implemented the methods of BIM, which is also known as bridge information modelling (BrIM) ^23^. Implementing BIM increases the likelihood of considerably improving the efficiency of projects, reducing waste, and improve sustainability throughout the life cycles of infrastructure projects ^24^. In bridge engineering, BIM has caused systematic and innovative changes, particularly for the design and construction stages of a project. Bridge lifecycle information can be gathered and managed by BIM in a way that is digital and consistent to standards ^25^. The conditions of a project can be evaluated throughout its life cycle with BIM. Furthermore, every information related to the project can be visualised and integrated in a way that is effective ^26^.

The health monitoring system of a bridge is made through the combination of BIM and traditional bridge health monitoring, which is capable of organising and visualising a significant amount of sensor data and subsequent structural health information over a lengthy period of time ^27^. BIM can be combined with the Internet of Things (IoT) tool to improve the smart management in a bridge life cycle. Thorough geometric and semantic information will be produced by BIM while Internet of Things has the management and analysis of the bridge’s true condition ^28^. There is a bridge health state, information integration, and safety warning management method that is BIM-based which uses Revit API interface to integrate the functional plug-ins used for monitoring information management and visual warning into the Revit software while using Revit software as a development platform ^29^. 6D BIM can be used for the lifecycle asset management of a bridge infrastructure by combining 3D model information with a time schedule, cost estimation, and carbon footprint analysis throughout the lifecycle of the bridge project ^30^. BIM could also be used for bridge rehabilitation and can be used to develop a method that involves the development and management of the digital representations of the physical and valuable features ^31^.

### Risks to bridges from climate change

The potential impacts of climate change include buckling of roads and railways caused by heat waves, and a higher risk of flooding ^32^. Hydraulic failure or scour is a usual event that causes bridge failure. In particular areas, climate change increases the risk of scour and the damage it causes. The speed of stream flows will become faster, leading to higher scour rates and higher temperatures. Additionally, snowmelt will cause an outcome of higher water levels that will also increase scour rates ^8^. The effects of global warming will cause great increase to hurricane-related hazards, particularly higher wind speeds combined with powerful storm surge and torrential rainfall in the upcoming years ^33^. The structural durability and the creep shrinkage of concrete bridges will worsen because of increased carbon dioxide concentration and increased temperatures that occur due to climate change. Additional loading that leads to the bridge overloading will also have a high negative impact on the reliability of existing bridges in regard to time ^34^.

There will be a massive cost for reconstructing damaged or collapsed structures if the impacts that are expected to be caused by climate change are not addressed sufficiently and proactively in the management, design, and construction of any upcoming transportation infrastructure project or the rehabilitation of any present existing infrastructure ^35^.

## Methods

There are different research methodologies that can be used. Analytical Research is a type of research method in which the researcher makes use of data and factual information available to them, and to interpret this information in order to begin a precise evaluation of the data. Fundamental Research is a research type that is mainly focused on formulating a theory. This type of research method seeks to find information using an extensive application base, and therefore, supplement the concepts that exists in a particular industry. Quantitative Research is a research method that involves the measurement of a specific amount or quantity of a certain event. It aims to collect and interpret numerical data and can also be used for finding any patterns or averages or for forming predictions. Qualitative research is a form of research that is concerned with the quality of a particular situation, it asks for the “why” and the “what”. With this research type, the factors that influence people to behave in a particular way or factors that control their preferences towards a specific thing can be discovered and interpreted. An Analytical Research Method is suitable for this research to meet the objectives ^36^.

The research methodology section will show all the processes that were necessary to develop the 6-dimensional model of a bridge that features climate change adaptation measures. Research to find possible climate change adaptation measures is done. From the research, seven climate change adaptation measures have been selected for the 6D BIM model. They were then added to the 3D model of a bridge previously created by 9 on Revit 2022 Software. From this model, a further 3 dimensions are developed to produce the climate change adaptation measures timeline schedule, climate change adaptation cost estimation, and carbon emission estimation. The 4D model is done on Navisworks Manage 2022 software. Similar packages are also embedded in alternative software such as Bentley BIM. The data for the Cost per kg and CO2 Footprint per kg of the materials required for the 5D model and 6D model is obtained from Granta EduPack. The University of Birmingham holds a copyright license for the use of Granta EduPack, allowing the incorporation of its data in academic research. The UoB Ethical Guidelines are followed during the research.

### Risk analysis

Before researching possible climate change adaptation measures, a risk analysis of the potential climate change impacts is carried out using a Risk Matrix. The climate change impacts will be ranked based on findings from the literature review. Table 1 shows the defined scale that will be used. The scale was then used to classify the climate change impacts, which is shown in Table 2. The Risk Matrix of the climate change impacts are shown in Table 3. Table 4 shows the adaptation measure chosen for each climate change impact, along with the required materials and benefits of the measure.

**Table 1.** Classification Scheme Scale.

Likelihood (L)		Consequence (C)	
Rank	Scale	Rank	Scale
5	Almost Certain	5	Serious
4	High	4	Significant
3	Medium	3	Moderate
2	Low	2	Minor
1	Rare	1	Insignificant

**Table 2.** Climate Chang Impact Classification.

Climate Change Impact Identification	Climate Change Impact	Likelihood Score	Consequence Score
1	Storms	4	5
2	Greater risk of stresses that are thermally caused	4	4
3	Damage to the pavement caused by heat	5	4
4	Floods	4	5
5	Additional snow load	3	3
6	Additional load on piles	5	4
7	Erosion	3	4

**Table 3.** Risk matrix of the climate change impacts.

**Likelihood (L)**	5				3,6		**Risk Level**
	4				2	1,4	High
	3			7	5		Medium
	2						Low
	1						
		1	2	3	4	5	
		**Consequence (C)**					

**Table 4.** Adaption measures selected for the model, their required materials, and benefits.

Adaption Measure	Climate change impact it will reduce the damage of	Required Materials	Benefits
Cladding added to the bridge	Storms	Glass Fiber Reinforced Polymer	Adding cladding to the bridge will provide a weather-resistant shield that protects the main structure from wind and weather impacts caused by storms ^37^.
Painting the bridge white	Greater risk of stresses that are thermally caused	Epoxies Paint	Painting the bridge white will lead to an albedo effect, which will lower the overheating of the bridge ^7^.
Using polymer modified binders	Damage to the pavement caused by heat	Bitumen Synthetic Polymer	Polymer Modified Binders are made up of bitumen mixed with a synthetic polymer. Polymer Modified Binders are used to improve the performance of binders on pavement surfaces that are distressed, mostly due to hostile climatic conditions. Furthermore, using Polymer Modified Binders will also increase its resilience and cohesion, reduce how susceptible it is to temperature, and also improve its tenacity ^38^.
Bridge Drainage System	Floods	Steel Polyvinylchloride (PVC)	Effectively and efficiently taking the water away from the surface of bridge with a suitable drainage system will decrease the risks of hydroplaning, protect the public and improve safety ^39^.
Jacketing the piers	Additional snow load	Steel Epoxy	Jacketing is a process that can be used to add or restore ultimate load capacity of the piers. It is used to help support additional live load or dead load that was not considered in the original design of the bridge ^40^.
Bitumen coating of the piles	Additional load on piles	Bitumen	Weatherproofing and corrosion resistance are the benefits that bituminous paint will give to the piles ^41^.
Applying shotcrete to the embankment	Erosion	Concrete	Shotcrete can be used to permanently cover the embankment that may eventually erode over time or deteriorate ^42^.

## Results

### 3D model

**Figure 3.**
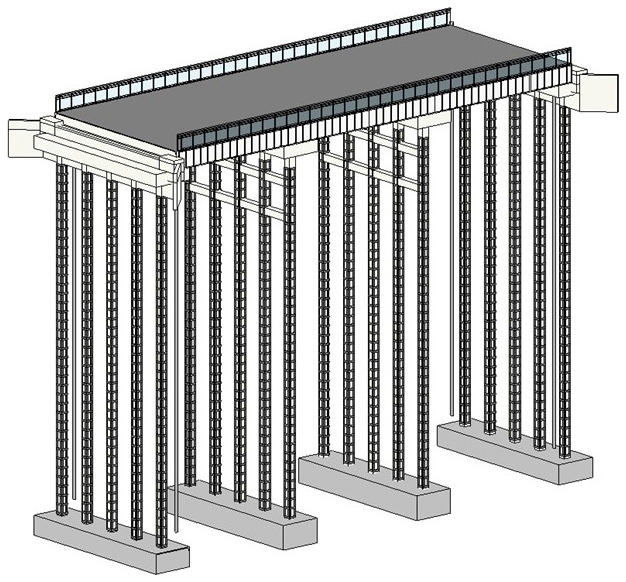
3D Model view of the bridge.

**Figure 4.**
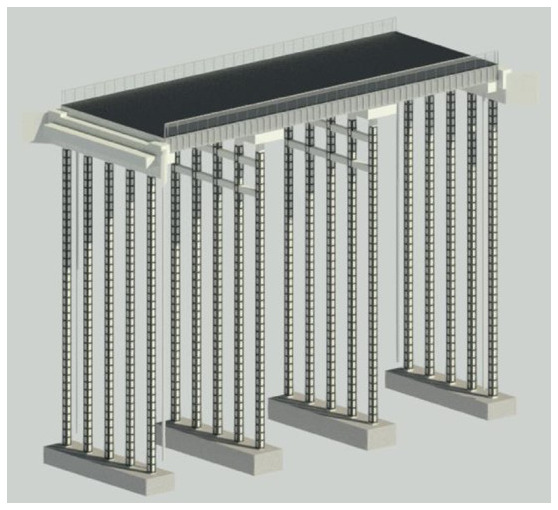
3D Render view of the bridge.

The adaption measures are added to the bridge on Revit 2022 software. Figure 3 shows the 3D model of the bridge and Figure 4 shows the 3D Render view of the bridge. The 3D model shows how the adaptation measures can be applied to a roadway width, slab bridge.

### 4D model: Timeline schedule

**Figure 5.**
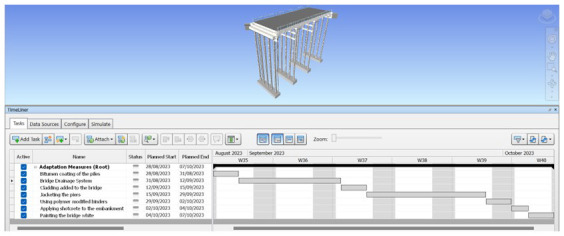
Complete Timeline of the bridge on Navisworks 2022.

**Figure 6.**
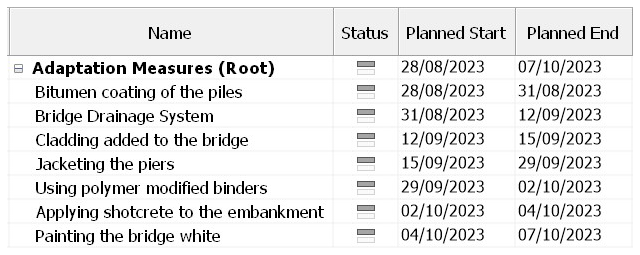
Zoomed In Timeline of the bridge.

After the adaptation measures have been successfully added to the 3D model, the 4D model is then created using Navisworks 2022 software. Figure 5 and Figure 6 show the estimated time schedule of the adaptation measures. It is assumed that no work is done during the weekends and as mentioned in the introduction, adaption measures can be implemented during the process of the construction of the bridge.

### 5D model: Cost estimation

**Figure 7.**
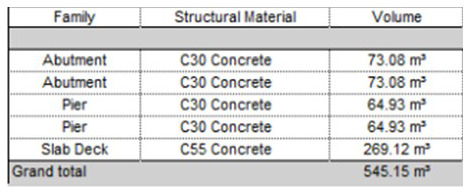
Dimensions of 3D bridge model.

**Figure 8.**
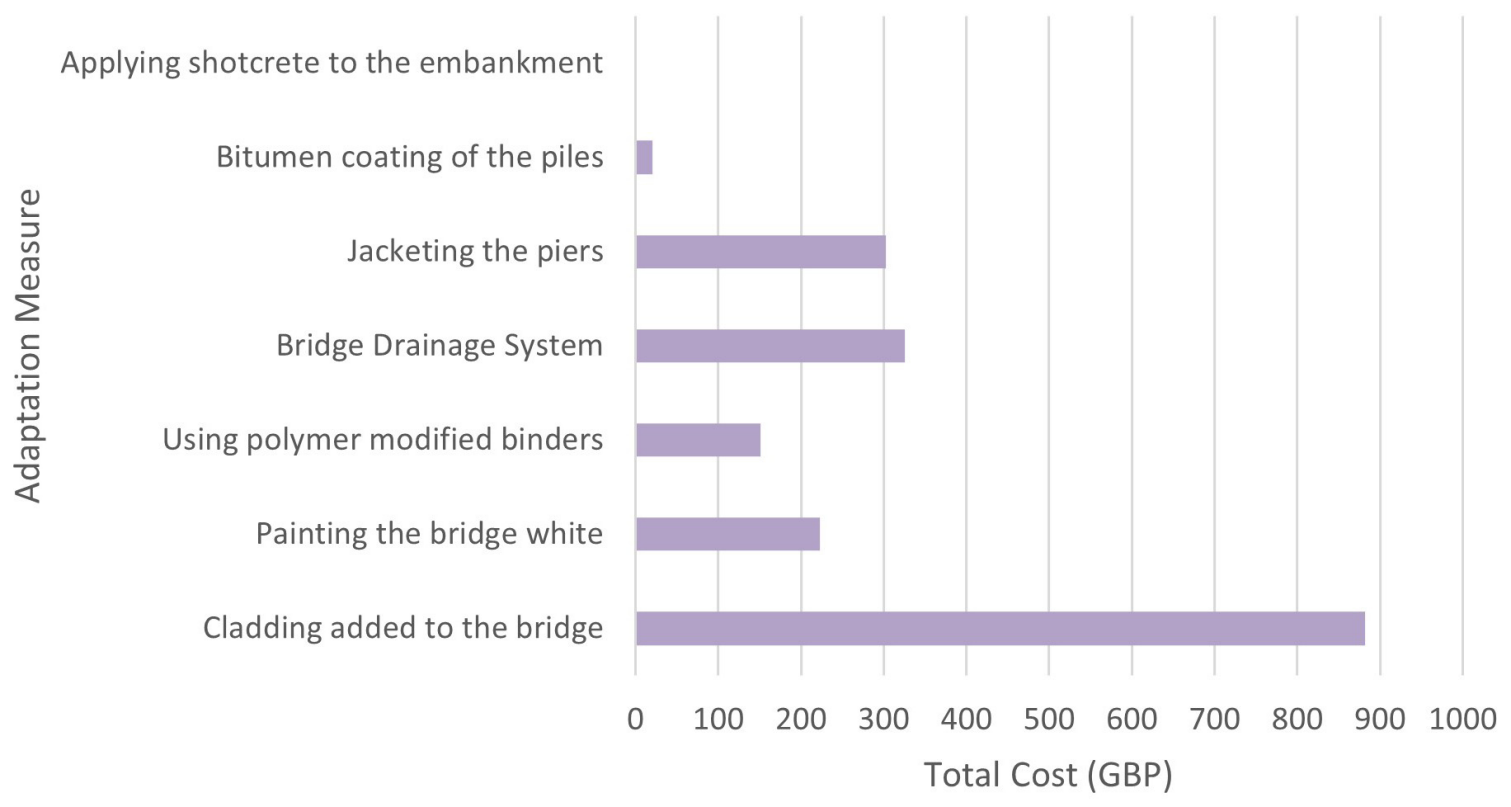
Graph of the total estimated cost of each Adaptation Measure.

After the 4D model has been completed, the 5D Model is then added. The mass used for the 5D model is estimated based on the dimensions of the 3D model bridge shown in Figure 7. Table 5 shows the total cost of each material needed for the adaption measures that is calculated with cost of each material obtained from Granta EduPack. The grand total cost of all the measures came out to be £1904.41. Figure 8 shows a graph of the total estimated cost of each adaptation measure.

**Table 5.** Cost Estimations of Adaptation Measures.

Adaptation Measure	Required Materials	Mass (kg)	Cost (GBP/Kg)	Total Cost (GBP)
Cladding added to the bridge	Glass Fiber Reinforced Polymer	354	2.49	881.46
Painting the bridge white	Epoxies Paint	70	3.19	223.30
Using polymer modified binders	Bitumen	135	0.27	36.45
	Synthetic Polymer	135	0.85	114.75
Bridge Drainage System	Steel	110	0.52	57.20
	Polyvinylchloride (PVC)	200	1.34	268.00
Jacketing the piers	Steel	275	0.52	143.00
	Epoxy	50	3.19	159.50
Bitumen coating of the piles	Bitumen	75	0.27	20.25
Applying shotcrete to the embankment	Concrete	25	0.04	1.00
Grand Total				**1904.41**

### 6D model: Carbon emission estimation

**Figure 9.**
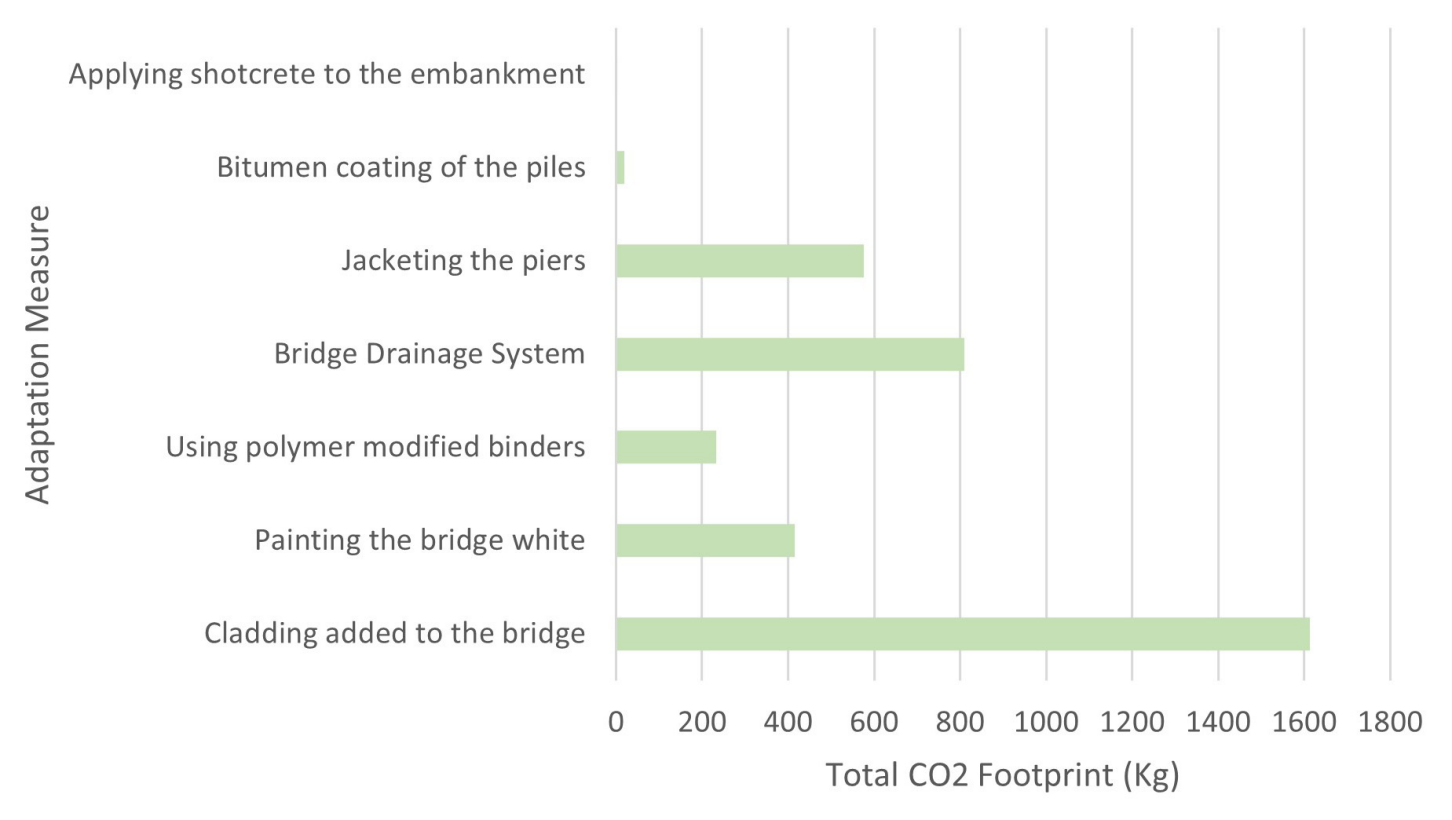
Graph of the total estimated carbon emission of each Adaptation Measure.

The 6D model is next after the completion the 5D model. As mentioned previously in the analysis of the 5D model, the mass used for the 6D model is estimated from the measurements of the previously created 3D model shown in Figure 7. Table 6 shows the grand total estimated CO2 footprint was 3670.64 kg. Figure 9 shows a graph of the total estimated carbon emission of each adaptation measure.

**Table 6.** Carbon Emission Estimation of the Adaptation Measures.

Adaptation Measure	Required Materials	Mass (kg)	CO2 Footprint (Kg/Kg)	Total CO2 Footprint (Kg)
Cladding added to the bridge	Glass Fiber Reinforced Polymer	354	4.56	1614.24
Painting the bridge white	Epoxies Paint	70	5.94	415.80
Using polymer modified binders	Bitumen	135	0.25	33.75
	Synthetic Polymer	135	1.47	198.45
Bridge Drainage System	Steel	110	2.33	256.30
	Polyvinylchloride (PVC)	200	2.77	554.00
Jacketing the piers	Steel	120	2.33	279.60
	Epoxy	50	5.94	297.00
Bitumen coating of the piles	Bitumen	75	0.25	18.75
Applying shotcrete to the embankment	Concrete	25	0.11	2.75
**Grand Total**				**3670.64**

## Discussion

The results from this project demonstrates how 6D BIM can be used to support the improvement of bridges to withstand the impacts of climate change. The 3D model shows that the measures can be applied to the bridge without hindering the main function of the bridge. The 4D model shows an estimated timeline schedule that brings benefits such as being able to foresee potential hazards, the enhancement of project planning, the improvement of communication, producing more collaboration and improving the visualisation of the project ^43^. The 5D model shows the total estimated cost of the adaptation measures, where “Cladding added to the bridge” has the highest estimated cost while “Applying shotcrete to the embankment” has the lowest estimated cost. 5D BIM enables stakeholders to understand, analyse, discover, and record the effects of modifications on the cost of the project ^44^. The 6D model shows the total estimated carbon emission of the adaptation measures, where “Cladding added to the bridge” has the highest estimated CO2 footprint while “Applying shotcrete to the embankment” has the lowest estimated CO2 footprint. Using 6D BIM leads to a prediction of energy consumption data that is precise, which then produces the possible cost of an asset that is in a project cycle. This supports the analysis of how the budget should be used to achieve sustainability and cost-efficiency that is done by the stakeholders ^45^.

## Conclusion

This study presents a comprehensive approach towards addressing the critical challenge of enhancing bridge resilience against the impacts of climate change through the utilization of Building Information Modelling (BIM). By employing a 6D BIM framework, encompassing a range of dimensions including the integration of climate change adaptation measures within a 3D Revit model, development of timeline schedules, cost estimations, and carbon emission assessments, the research underscores the effectiveness of BIM in bolstering bridge infrastructure resilience. The results demonstrate that the integration of adaptation measures does not compromise the functionality of the bridge, as evidenced by the seamless incorporation depicted in the 3D model. Moreover, the timeline scheduling facilitated by the 4D model enhances hazard anticipation, project planning, communication, collaboration, and project visualization. Cost estimations from the 5D model reveal diverse cost profiles among adaptation measures, while the 6D model provides insights into varying carbon footprints. These dimensions of BIM enable stakeholders to analyze and optimize project costs and energy consumption, thereby fostering sustainability and cost-efficiency considerations throughout the project lifecycle. Overall, this research contributes valuable insights and practical implications for enhancing bridge resilience globally, providing a robust foundation for future endeavors in climate change adaptation within bridge engineering.

### Limitations

The bridge was assumed to be located in the UK, which has certain weather conditions that is different to other parts of the world. So, the adaption measures may need to be adapted to the type of weather of the country that the bridge is located in.

### Further work

This project can be improved in future work by also considering the equipment required and processes of the adaption measures when developing the 6D BIM model, particularly for the cost estimation and the carbon emission estimation. This will help the long-term goal of the construction industry to reduce the negative impacts that climate change can potentially cause to bridges around the world.

## Ethics and consent

Ethical approval and written informed consent were not required.

## Data Availability

Zenodo: Dataset for digital twins for managing bridge climate change adaptation, https://doi.org/10.5281/zenodo.12532481 ^46^ This project contains the following underlying data: - Project Calculation.xlsx. (detail of cost and carbon emission calculation) Data are available under the terms of the Creative Commons Attribution 4.0 International license (CC-BY 4.0). Other data can be made available upon appropriate request to the Department of Highway, Thailand. Any data request will be required to seek further written permission from the Department of Highway (Address: 2/486 Thanon Si Ayutthaya, Thung Phaya Thai, Ratchathewi, Bangkok 10400, Thailand; E-mail: saraban@doh.go.th).
