# Supplementary material for: Digital twins for managing bridge climate change adaptation
Source: Open Res Eur. 2024 Sep 26;4:173. Originally published 2024 Aug 8. [Version 2] doi: 10.12688/openreseurope.17809.2 (PMC11437294; doi:10.12688/openreseurope.17809.2)
Supplement: Peer review history

# Reviewer report: Reviewer response for version 2

**DOI:** 10.21956/openreseurope.20058.r44663

**Published:** 2024-09-27

**Authors:** Sih Ying Kong

**Affiliations:** 1 Civil, Monash University Malaysia, Bandar Sunway, Selangor, Malaysia; 2 Civil, Monash University, Subang Jaya, Selangor, Malaysia

No further comments to make

Is the study design appropriate and does the work have academic merit?

Yes

Is the work clearly and accurately presented and does it cite the current literature?

Partly

If applicable, is the statistical analysis and its interpretation appropriate?

Not applicable

Are all the source data underlying the results available to ensure full reproducibility?

Yes

Are the conclusions drawn adequately supported by the results?

Yes

Are sufficient details of methods and analysis provided to allow replication by others?

Partly

Reviewer Expertise:

Structures

I confirm that I have read this submission and believe that I have an appropriate level of expertise to confirm that it is of an acceptable scientific standard.

# Reviewer report: Reviewer response for version 1

**DOI:** 10.21956/openreseurope.19252.r42975

**Published:** 2024-08-23

**Authors:** Salvatore Antonio Biancardo

**Affiliations:** 1 University of Naples Federico II, Napoli,, Italy; 2 Universita degli Studi di Napoli Federico II Dipartimento di Ingegneria Civile Edile e Ambientale, Naples, Campania, Italy

The manuscript presents a comprehensive approach to addressing the critical challenge of enhancing bridge resilience against the impacts of climate change through the use of BIM.

 The manuscript is well-written and structured. Limitations of the study and future works are well presented.

 The readability of Figure 7 should be improved.

Is the study design appropriate and does the work have academic merit?

Yes

Is the work clearly and accurately presented and does it cite the current literature?

Yes

If applicable, is the statistical analysis and its interpretation appropriate?

Yes

Are all the source data underlying the results available to ensure full reproducibility?

Yes

Are the conclusions drawn adequately supported by the results?

Yes

Are sufficient details of methods and analysis provided to allow replication by others?

Yes

Reviewer Expertise:

BIM for infrastructure

I confirm that I have read this submission and believe that I have an appropriate level of expertise to confirm that it is of an acceptable scientific standard.

# Reviewer report: Reviewer response for version 1

**DOI:** 10.21956/openreseurope.19252.r42972

**Published:** 2024-08-14

**Authors:** Sih Ying Kong

**Affiliations:** 1 Civil, Monash University Malaysia, Bandar Sunway, Selangor, Malaysia; 2 Civil, Monash University, Subang Jaya, Selangor, Malaysia

The paper presents a 6D BIM framework used to enhance bridge resilience against climate change impacts. The modelling info of each stage is described to allow the reproduction of the results. The idea is interesting, the writing can be improved. The detailed comments are listed below:

1. Lacking elaboration for Tables 1 to 3. It is not clear how the scores of likelihood and consequence presented in Table 2 were obtained. Clear justifications for the score will be helpful for the readers to relate the climate change impacts on bridges.
2. Figure 3 and Figure 4 seem to repeat the same info. The authors may consider deleting either one. It is recommended to label important components in the figure for clarity.
3. It is stated in the introduction that it is assumed the bridge is not constructed yet. But the timeline shown in Figure 6 shows all the adaption is completed in 2023. It will be good to revise the timeline to align with the assumption stated in the introduction
4. The text in Figures 5, 6 and 7 is not clear. Please improve the quality of these figures.
5. the cost of 1 GBP for concrete in Table 5 seems very low. Please check and explain why it is so low
6. Typos could be observed in the paper. It is recommended to proofread the paper

Is the study design appropriate and does the work have academic merit?

Yes

Is the work clearly and accurately presented and does it cite the current literature?

Partly

If applicable, is the statistical analysis and its interpretation appropriate?

Not applicable

Are all the source data underlying the results available to ensure full reproducibility?

Yes

Are the conclusions drawn adequately supported by the results?

Yes

Are sufficient details of methods and analysis provided to allow replication by others?

Partly

Reviewer Expertise:

Structures

I confirm that I have read this submission and believe that I have an appropriate level of expertise to confirm that it is of an acceptable scientific standard, however I have significant reservations, as outlined above.

# Response

**Published:** 2024-09-09

**Authors:** Sakdirat Kaewunruen

**Affiliations:** University of Birmingham, UK

The authors appreciate the constructive and kind comments provided by the expert reviewer. Your comments have been fully appended in the revision.  

**Reviewer Comments:**

 The paper presents a 6D BIM framework used to enhance bridge resilience against climate change impacts. The modelling info of each stage is described to allow the reproduction of the results. The idea is interesting, the writing can be improved. The detailed comments are listed below:  

 1. Lacking elaboration for Tables 1 to 3. It is not clear how the scores of likelihood and consequence presented in Table 2 were obtained. Clear justifications for the score will be helpful for the readers to relate the climate change impacts on bridges.

**Author Reply:** Thank you for your valuable feedback. I have now provided more detailed explanations for the data presented in Tables 1 to 3, specifically clarifying the methodology used to derive the likelihood and consequence scores in Table 2. I believe these justifications will help readers better understand how the scores relate to climate change impacts on bridges.    

**Reviewer Comments:**

 2. Figure 3 and Figure 4 seem to repeat the same info. The authors may consider deleting either one. It is recommended to label important components in the figure for clarity.

**Author Reply:**  Figure 4 has been removed, and the key components of the bridge in Figure 3 have been clearly labelled for improved clarity.  

**Reviewer Comments:**

 3. It is stated in the introduction that it is assumed the bridge is not constructed yet. But the timeline shown in Figure 6 shows all the adaption is completed in 2023. It will be good to revise the timeline to align with the assumption stated in the introduction

**Author Reply:** The timeline has been revised to align with the assumption in the introduction, and the completion date has been adjusted to 2025.    

**Reviewer Comments:**

 4. The text in Figures 5, 6 and 7 is not clear. Please improve the quality of these figures.

**Author Reply:** The quality of Figures 5, 6, and 7 has been enhanced, and the text has been made clearer.    

**Reviewer Comments:**

 5. the cost of 1 GBP for concrete in Table 5 seems very low. Please check and explain why it is so low

**Author Reply:** The price is correct. The price of C30 concrete is around 40 pounds per ton. The usage of concrete is about 25kg, the price is only 1kg if only considering the material usage. An explanation has been added to the manuscript accordingly.  

**Reviewer Comments:**

 6. Typos could be observed in the paper. It is recommended to proofread the paper.

**Author Reply: **Thanks for the reviewer's comments. The manuscript has been thoroughly proofread and revised accordingly.

 The authors appreciate the constructive and kind comments provided by the expert reviewer. Your comments have been fully appended in the revision.
